# Magnetically actuated sonodynamic nanorobot collectives for potentiated ovarian cancer therapy

**DOI:** 10.3389/fbioe.2024.1374423

**Published:** 2024-03-26

**Authors:** Yixuan Zhou, Ziqi Cao, Lixian Jiang, Ying Chen, Xiaoyu Cui, Jianrong Wu, Xue Xie, Longchen Wang, Tao Ying

**Affiliations:** ^1^ Jinzhou Medical University Graduate Training Base (Shanghai Sixth People’s Hospital Affiliated to Shanghai Jiao Tong University School of Medicine), Jinzhou, China; ^2^ Department of Ultrasound in Medicine, Shanghai Sixth People’s Hospital Affiliated to Shanghai Jiao Tong University School of Medicine, Shanghai Institute of Ultrasound in Medicine, Shanghai, China

**Keywords:** magnetic nanorobot, sonodynamic therapy, ovarian cancer, drug delivery, magnetic nanoparticle

## Abstract

Ovarian cancer presents a substantial challenge due to its high mortality and recurrence rates among gynecological tumors. Existing clinical chemotherapy treatments are notably limited by drug resistance and systemic toxic side effects caused by off target drugs. Sonodynamic therapy (SDT) has emerged as a promising approach in cancer treatment, motivating researchers to explore synergistic combinations with other therapies for enhanced efficacy. In this study, we developed magnetic sonodynamic nanorobot (Fe_3_O_4_@SiO_2_-Ce6, FSC) by applying a SiO_2_ coating onto Fe_3_O_4_ nanoparticle, followed by coupling with the sonosensitizer Ce6. The magnetic FSC nanorobot collectives could gather at fixed point and actively move to target site regulated by magnetic field. *In vitro* experiments revealed that the magnetic FSC nanorobot collectives enabled directional navigation to the tumor cell area under guidance. Furthermore, under low-intensity ultrasonic stimulation, FSC nanorobot collectives mediated sonodynamic therapy exhibited remarkable anti-tumor performance. These findings suggest that magnetically actuated sonodynamic nanorobot collectives hold promising potential for application in target cancer therapy.

## 1 Introduction

Ovarian cancer is one of the most prevalent malignant neoplasms affecting the female reproductive system, and its global incidence is exceeded only by those of cervical and endometrial cancers (Torre et al., 2018; Sung et al., 2021). Most individuals with ovarian cancer are diagnosed at an advanced stage, leading to a 5-year survival rate of less than 30% (Srivastava et al., 2017; Sung et al., 2021). The therapeutic repertoire for ovarian cancer encompasses surgical intervention, chemotherapy, and radiotherapy (Kengsakul et al., 2022). Nevertheless, these modalities inevitably have some inherent limitations. To maximize lesion eradication, extensive radical surgery is frequently performed. Additionally, surgery may lead to peritoneal or abdominal lymph node metastasis, increasing postoperative complications and mortality (Gerestein et al., 2009; Di Donato et al., 2017). Chemotherapy is a cornerstone of treatment but is burdened by potent toxic side effects, drug resistance and consequently decreased treatment efficacy (Coleman et al., 2013). Radiotherapy, which is applicable only to a subset of ovarian cancer patients, poses challenges in achieving a curative effect, with repeated sessions potentially diminishing progression-free survival (Durno and Powell, 2022). Despite ongoing refinements in traditional treatment methodologies, including surgery, chemotherapy, and radiotherapy, the efficacy of these approaches remains suboptimal (Giovanni et al., 2018). Therefore, it is imperative to explore novel therapeutic strategies for the effective management of ovarian cancer.

In recent years, sonodynamic therapy (SDT), an emerging noninvasive modality for tumour treatment, has exhibited substantial promise in cancer therapy owing to its heightened efficacy, deep tissue penetration, elevated selectivity, and minimal impact on surrounding normal tissues (McHale et al., 2016; Qian et al., 2016; Pan et al., 2018; Yan et al., 2020; Yang et al., 2021; Wang D. et al., 2022; Wang J. et al., 2023). SDT is based on the combination of ultrasound and sonosensitizers: low-intensity ultrasound is used to stimulate sonosensitizers that have been enriched in deep tumor tissues (Wang et al., 2020; Wang et al., 2021a; Ning et al., 2022; Guo et al., 2023). This activation elicits the generation of reactive oxygen species (ROS), thereby inducing apoptosis in tumor cells (Guo et al., 2022). Widely employed sonosensitizers include porphyrins and their derivatives (such as hematoporphyrin and protoporphyrin), porphyrin compounds (such as dihydroxyacetone-porphyrin e6), inorganic nanomaterials (including titanium dioxide, zinc oxide, and ferric oxide tetroxide), hybrids thereof (Wang et al., 2021b), and metal-based systems (Zhang et al., 2018; Zhang et al., 2019; Son et al., 2020). Notably, dihydroxyacetone-porphyrin e6 (Ce6), as an exemplary sonosensitizer, manifests robust acoustic activity, a high quantum yield of singlet oxygen production, and exceptional penetration capabilities, and it is therefore widely utilized (Sun et al., 2017; Wang S. et al., 2022; Zhu et al., 2022; Chen et al., 2023). However, the dose of the sonosensitizer arriving at the tumor area is very limited due to the lack of active targeting capability. In addition, owing to hemodynamics and the interstitial fluid pressure of tumors, the passive targeting efficiency of systemically administered sonosensitizer drugs to tumor tissues is further diminished. Consequently, the pursuit of a strategy that can precisely target tumor sites and increase the efficiency of sonosensitizer accumulation within tumors has become an imperative research objective.

To address the limited targeting efficiency inherent in conventional therapeutic approaches, researchers have devised a multitude of nanocarriers that leverage the altered cellular properties of cancer cells. These nanocarriers are intricately coupled with targeting ligands, such as peptides, proteins, antibodies, or aptamers, which are designed to recognize specific surface markers on cancer cells. The primary objective of this strategy is to extend the drug half-life and increase the precision of tumor-targeting capabilities (Shi et al., 2017). Despite encouraging prospects, an expanding body of evidence indicates that, on average, only approximately 0.7% of nanocarriers successfully reach solid tumors, which significantly limits the clinical efficacy of current nanocarrier technologies (Wilhelm et al., 2016).

Magnetically actuated micro/nanorobots provide a promising and innovative paradigm for drug delivery (Li et al., 2017; Wang et al., 2021c). These systems amalgamate the advantages of previous nanomedicines, which are characterized by high selectivity and biocompatibility, with active and controllable locomotion capabilities. This integration facilitates the controlled delivery of nanomedicine directly to the tumor region. Compared with traditional targeted delivery methods, magnetically actuated micro/nanorobots demonstrate elevated precision in targeting and increasing the utilization of administered drugs (Hu et al., 2020; Schmidt et al., 2020; Zhou et al., 2021; Nguyen et al., 2023). Our research group has previously developed various magnetic actuation systems and validated drug delivery methods for the treatment of various diseases, such as thrombus therapy, osteoarthritis treatment and anti-infection therapy (Wang et al., 2021d; Ma et al., 2022; Tang et al., 2022; Zhao et al., 2022; Wang L. et al., 2023). Therefore, it is reasonable to believe that the sonosensitizer can be actively delivered to the tumor site to achieve enrichment by magnetic nanorobots.

**SCHEME 1:**
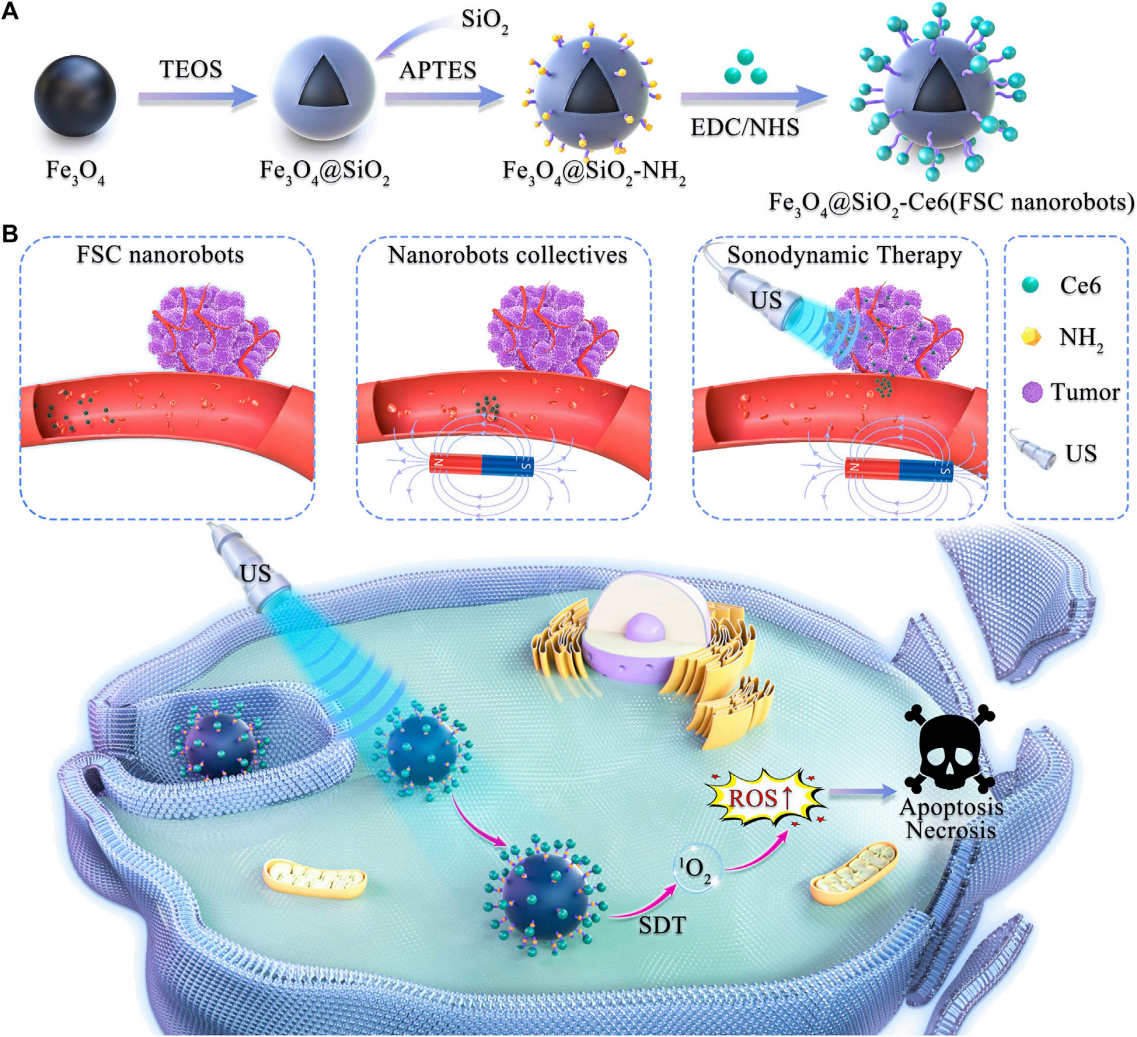
Schematic illustration of magnetically actuated sonodynamic nanorobot collectives to potentiate ovarian cancer therapy. **(A)** Synthetic procedure for the magnetic Fe3O4@SiO2-Ce6 nanorobot (FSC nanorobot). **(B)** Magnetically actuated sonodynamic nanorobot collectives to potentiate ovarian cancer therapy. FSC nanorobot collectives actively move to the tumor site via actuation by a magnetic field, which enables sonosensitizer delivery. Under low-intensity ultrasound irradiation, sonosensitizers loaded with nanorobot collectives can generate reactive oxygen species (ROS) to increase the cytotoxic effect on tumor cells.

Herein, a magnetically actuated sonodynamic nanorobot drug delivery system employing an Fe_3_O_4_@SiO_2_-Ce6 (FSC) nanorobot and magnetic actuation system was devised for targeted tumor treatment (Scheme 1). The FSC nanorobot consists of magnetic Fe_3_O_4_@SiO_2_-NH_2_ nanomaterials coupled with the sonosensitizer Ce6 (Fe_3_O_4_@SiO_2_-Ce6). Under the guidance of a magnetic field, FSC nanorobots can be aggregated to collectives and actuated to move to the target site, resulting in the active delivery of the sonosensitizer to the tumor site. This process amplifies the effect of SDT facilitated by Ce6, generating ROS to increase the cytotoxic impact on tumor cells. The validation of these outcomes involved characterization of the nanorobots, assessment of the sonodynamic ROS production, locomotion of the magnetic nanorobot collectives, and *in vitro* ovarian cancer cell therapy. The aim of this study was to explore the efficacy of magnetically actuated sonodynamic nanorobot collectives for ovarian cancer therapy and to further expand their application prospects in other tumor treatments.

## 2 Materials and methods

### 2.1 Synthesis of FSC nanorobots

First, Fe_3_O_4_ (0.1 g) was dispersed in ethanol (60 mL), deionized water (10 mL) and ammonium hydroxide solution (1.0 mL) and sonicated for 40 min. Tetraethyl orthosilicate (2.0 mL) was added, and the mixture was stirred for 24 h to obtain Fe_3_O_4_@SiO_2_. Fe_3_O_4_@SiO_2_ (20 mg) was dispersed in ethanol solution and functionalized with 3-aminopropyltriethoxysilane (APTES, 1.5 mL) for 24 h. The precipitate was collected with a magnet and washed several times to obtain Fe_3_O_4_@SiO_2_-NH_2_. For Ce6 coupling, Ce6 (4.0 mg), EDC (23.0 mg) and NHS (13.8 mg) we. re dissolved in dimethyl sulfoxide (4 mL), and Fe_3_O_4_@SiO_2_-NH_2_ (16 mL, 50 mg mL^-1^) was added to the mixed solution and stirred for 24 h. The reaction products were separated with a magnet and washed several times to obtain Fe_3_O_4_@SiO_2_-Ce6 (FSC) for further use.

### 2.2 Characterization of FSC nanorobots

The microstructure of the FSC nanorobots was observed by transmission electron microscopy (TEM) on a JEM-2100F instrument. The valence states and elemental composition of the FSC nanorobots were characterized by X-ray photoelectron spectroscopy (XPS, ESCAlab250 instrument) and Fourier transform infrared spectroscopy (FTIR), respectively. The potential and hydrodynamic particle size were measured by dynamic laser scattering on a Zeta Sizer system (Nano ZS90, Malvern Instruments Ltd.). The magnetic properties were evaluated by a vibrating sample magnetometer. The UV‒vis–NIR absorption spectra of the Fe_3_O_4_@SiO_2_-NH_2_, Ce6 and Fe_3_O_4_@SiO_2_-Ce6 nanoparticles were measured with a Shimadzu UV-3600 ultraviolet‒visible spectrophotometer.

### 2.3 *In vitro* ROS assay

DPBF experiment: A DMF solution (8 mM, 40 μL) containing DPBF with an FSC nanorobot dispersion (150 μg mL^-1^, 2.96 mL) was prepared and irradiated with low-intensity ultrasound (1.0 MHz, 1.0 W cm^-2^, 50% duty cycle), and the change in the absorption value at 419 nm was measured every 2 min with an ultraviolet spectrophotometer for 10 min in total. ESR experiment: TEMP (200 mM, 20 μL) was added to the FSC nanorobot dispersion (150 μg mL^-1^, 20 μL) and Ce6 solution (10 μg mL^-1^, 20 μL). After ultrasound irradiation (1.0 MHz, 1.0 W cm^-2^, 50% duty cycle, 2 min), the ^1^O₂ signal was immediately detected by ESR. For control purposes, the Ce6+TEMP, Ce6+TEMP + US, FSC + TEMP, and FSC + TEMP + US groups were tested simultaneously.

### 2.4 Magnetic locomotion of FSC nanorobot collectives

The magnetic nanorobot dispersion (2 mg mL^-1^, 1 mL) was placed in a confocal dish on the platform of the magnetic actuation system. Then, a rotating magnetic field (2 Hz) was applied to make the nanorobots aggregate at one point. For the locomotion of the nanorobot collectives, different biological fluids were deployed in the custom-designed ‘R’, ‘O’, and ‘S’ channels of acrylic plates. The magnetic nanorobot dispersion (2 mg mL^-1^, 20 μL) was added to the channels separately. By adjusting the moving platform, the magnetic nanorobot could be directed along a trajectory. The images were recorded by an operating microscope (DOM-1001, RWD Life Science, Shenzhen, China).

### 2.5 Cell culture and *in vitro* cytotoxicity assay

Mouse ovarian epithelial cancer cells (ID8) (Shanghai Institute of Cells, Chinese Academy of Sciences) were precultured in 96-well plates (1 × 10^4^ cells/well) for 12 h and allowed to adhere. The medium was then removed from each well, and the cells were washed twice with phosphate buffer (PBS). Subsequently, Fe_3_O_4_@SiO_2_-Ce6 nanoparticles at different concentrations (0, 100, 200, 400 and 800 μg mL^-1^) were dispersed in high-glucose DMEM containing 10% foetal bovine serum (FBS), added to each well, and incubated with the cells for 24 h and 48 h, respectively. The cells were then washed with PBS, and a standard CCK-8 assay (100 μL, V_CCK8_: V_DMEM_ = 1: 9) was performed to detect cell viability, which was measured on a microplate reader at a wavelength of 450 nm after incubation for 1–2 h.

### 2.6 Intracellular ROS assay

ID8 cells (1 × 10³ cells/well) were seeded into confocal-specific plates overnight. After the cells had completely adhered to the wall, the cell medium was replaced, and the cells were treated with Fe_3_O_4_@SiO_2_ and FSC for 12 h. Subsequently, the cells were further exposed to US irradiation (1.0 MHz, 1.0 W cm^-2^, 50% duty cycle, 5 min) and incubated with DCFH-DA (1:1,000 dilution) for another 30 min. Then, the treated cells were washed 3 times with PBS and imaged by fluorescence microscopy. Correspondingly, the cells were collected and analysed quantitatively for intracellular green fluorescence intensity by flow cytometry (FACSCalibur; BD Biosciences).

### 2.7 Intracellular GSH and GSSG assay

ID8 cells were seeded in several 6-well plates for overnight. After that, the cells were incubated with Fe_3_O_4_@SiO_2_ and FSC (400 μg mL^-1^) for 12 h. Next, the cells were stimulated with or without US (1.0 Wcm^-2^, 1.0 MHz, 50% duty cycle) for 5 min. After that, the cells were washed with PBS, and were frozen and thawed 3 times. The liquid supernatants were collected for GSH/GSSG detection using the assay kit.

### 2.8 Intracellular BCA and MDA assay

ID8 cells were seeded in culture dishes for overnight and randomly divided into four groups (control, Fe_3_O_4_@SiO_2_, FSC, FSC + US). The cells were cultured for 12 h and then subjected to US irradiation for 5 min. Subsequently, the cells were rinsed with PBS. Cell samples treated for quantitative protein analysis with a BCA protein assay kit. Then, the MDA assay kit reagents were added according to the manufacturer’s instructions, and the mixtures were heated in a boiling water bath at 100 °C for 15 min. After centrifugation at 12,000 rpm for 10 min, the absorbance of the supernatant was measured with a microplate reader at a wavelength of 532 nm. The MDA content in each sample was calculated in terms of the unit weight of protein content, and the results are presented as μmol MDA/mg protein.

### 2.9 Efficacy of SDT with magnetic actuation of FSC nanorobot collectives

ID8 cells were seeded in 6-well plates (1 × 10^5^/well) and cultured for 24 h. The cells were divided into the following experimental groups: ① simple diffusion + US group; ② magnetic regulation + US group. For the simple diffusion + US group, the FSC nanorobot dispersion was applied to the nontumor cell area in the middle of the round dish and allowed to freely disperse to the tumour cell area. In the magnetic regulation + US group, the FSC nanorobot dispersion was also applied to the nontumor cell area in the middle of the dish, and the dispersion was then aggregated by a magnetic actuation system and directed to the tumour cell area. US irradiation (1.0 MHz, 1.0 W cm^-2^, 50% duty cycle, 5 min) was applied to the tumour cell area, and the cells were incubated for 12 h. All treated cells were digested with trypsin and stained with the Calcein-AM/PI Kit for fluorescence microscopy.

### 2.10 Statistical analysis

For all the experiments conducted, we ensured that there were at least three replicates included. The data obtained from these experiments were presented as the mean value along with the standard error of the mean (SEM). To assess the differences between two groups, we employed the independent sample t-test. Additionally, for comparisons among multiple groups, we used one-way analysis of variance (ANOVA) followed by the Tukey post-test. In our analysis, statistical significance was denoted as *p* < 0.05, and this was further specified as *p < 0.05, **p < 0.01, ***p < 0.001.

## 3 Results and discussion

### 3.1 Synthesis and characterization of FSC nanorobot

**FIGURE 1:**
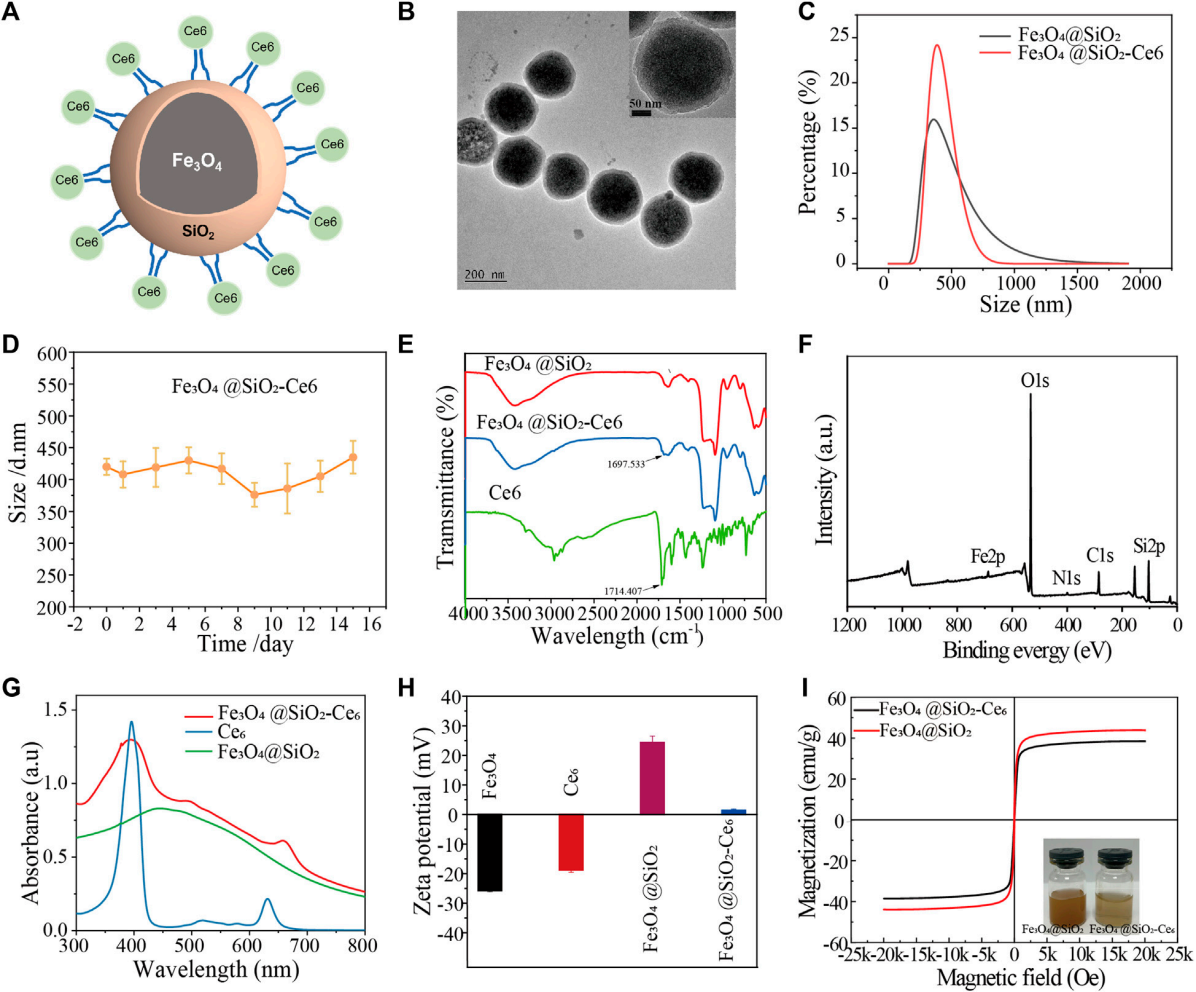
Structural characterization of Fe_3_O_4_@SiO_2_-Ce6 (FSC nanorobots). **(A)** Structure diagram of Fe_3_O_4_@SiO_2_-Ce6. **(B)** TEM images of Fe_3_O_4_@SiO_2_-Ce6 nanoparticles. **(C)** Hydrodynamic size of Fe_3_O_4_@SiO_2_ and Fe_3_O_4_@SiO_2_-Ce6. **(D)** Hydrodynamic size change of Fe_3_O_4_@SiO_2_-Ce6 dispersed in saline for 15 days. **(E)** FTIR spectra of Ce6, Fe_3_O_4_@SiO_2,_ and Fe_3_O_4_@SiO_2_-Ce6. **(F)** XPS spectra of Fe_3_O_4_@SiO_2_-Ce6. **(G)** UV‒Vis-NIR spectra of Ce6, Fe_3_O_4_@SiO_2,_ and Fe_3_O_4_@SiO_2_-Ce6. **(H)** Zeta potentials of Fe_3_O_4,_ Ce6, Fe_3_O_4_@SiO_2,_ and Fe_3_O_4_@SiO_2_-Ce6. **(I)** Vibrating magnetization curves of Fe_3_O_4_@SiO_2_ and Fe_3_O_4_@SiO_2_-Ce6. The insets show photographs of the Fe_3_O_4_@SiO_2_ and Fe_3_O_4_@SiO_2_-Ce6 solutions.

A structural diagram of an FSC nanorobot is shown in Figure 1A. TEM images illustrate that the FSC nanorobots had robust spherical morphology, uniform size, and good dispersibility (Figure 1B). The average hydrodynamic size was approximately 460 nm (Figure 1C), with no significant change observed after half a month in a physiological environment, indicating excellent dispersion stability (Figure 1D). Fourier transform infrared spectroscopy revealed a characteristic peak at 1,697.533 cm^-1^ for the -C=O of Ce6 in FSC (Figure 1E). X-ray photoelectron spectroscopy (XPS) analysis was conducted to examine the chemical composition and state of FSC. As shown in Figure 1F, obvious diffraction peaks were observed at 711.3, 532.5, 399.3, 284.6 and 103.3 eV, which can be attributed to Fe2p, O1s, N1s, C1s and Si2p, providing further confirmation that the FSC consist of Fe, O, N, C, and Si. The UV absorption spectrum of FSC exhibited characteristic absorption peaks of Ce6 at 401 nm and 656 nm, confirming successful coupling (Figure 1G). The potential of Fe_3_O_4_@SiO_2_ was 24.30 ± 2.2 mV. Upon coupling with Ce6, the potential of FSC changed to 1.40 ± 0.39 mV (Figure 1H), indicating that Ce6 was coupled with Fe_3_O_4_@SiO_2_. The vibrating magnetization curve results demonstrated that FSC exhibited a saturation magnetization of 38.45 emu g-^1^ in the high-field region of 2000 Oe, facilitating effective magnetic separation under the influence of an applied magnetic field and confirming its desirable superparamagnetic properties (Figure 1I).

### 3.2 *In Vitro* SDT effect of FSC nanorobot

**FIGURE 2:**
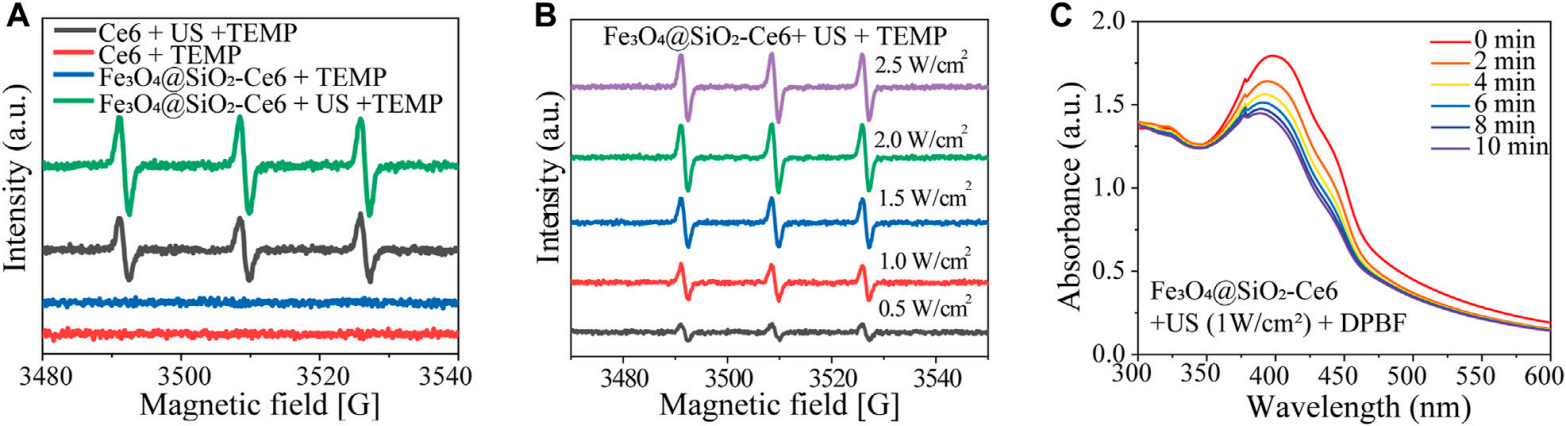
Sonodynamic properties of Fe_3_O_4_@SiO_2_-Ce6 (FSC nanorobots). **(A)** ESR signals of TEMP at different solutions. **(B)** ESR of Fe_3_O_4_@SiO_2_-Ce6 solution irradiated by US (different powers). **(C)** UV−vis spectra of Fe_3_O_4_@SiO_2_-Ce6 solution containing DPBF and further exposed to US irradiation (power density, 1.0 Wcm^−2^; duty cycle: 50%) for different durations (0, 2, 4, 6, 8 and 10 min).

We further examined the *in vitro* ROS production of Fe_3_O_4_@SiO_2_-Ce6 (FSC nanorobot). The quantity of ROS in the four groups was analyzed. The ESR spectra (Figure 2A) showed significant ^1^O_2_ generation under US irradiation in the Ce6 + US group and the FSC nanorobot + US group compared to the groups not subjected to US irradiation. Moreover, within a specific concentration range, the production efficiency of ^1^O_2_ was found to be correlated with the power intensity of US (Figure 2B). Simultaneously, DPBF acts as a ROS probe: the generated ROS can react with DPBF, resulting in a decrease in the characteristic absorption at 410 nm in the UV‒Vis spectrum. The absorption peak of DPBF decreased with time under US irradiation (1.0 MHz, 1.0 W cm^-2^, 50% duty cycle), indicating favorable ROS generation performance (Figure 2C).

### 3.3 Targeted locomotion of magnetic FSC nanorobot collectives

**FIGURE 3:**
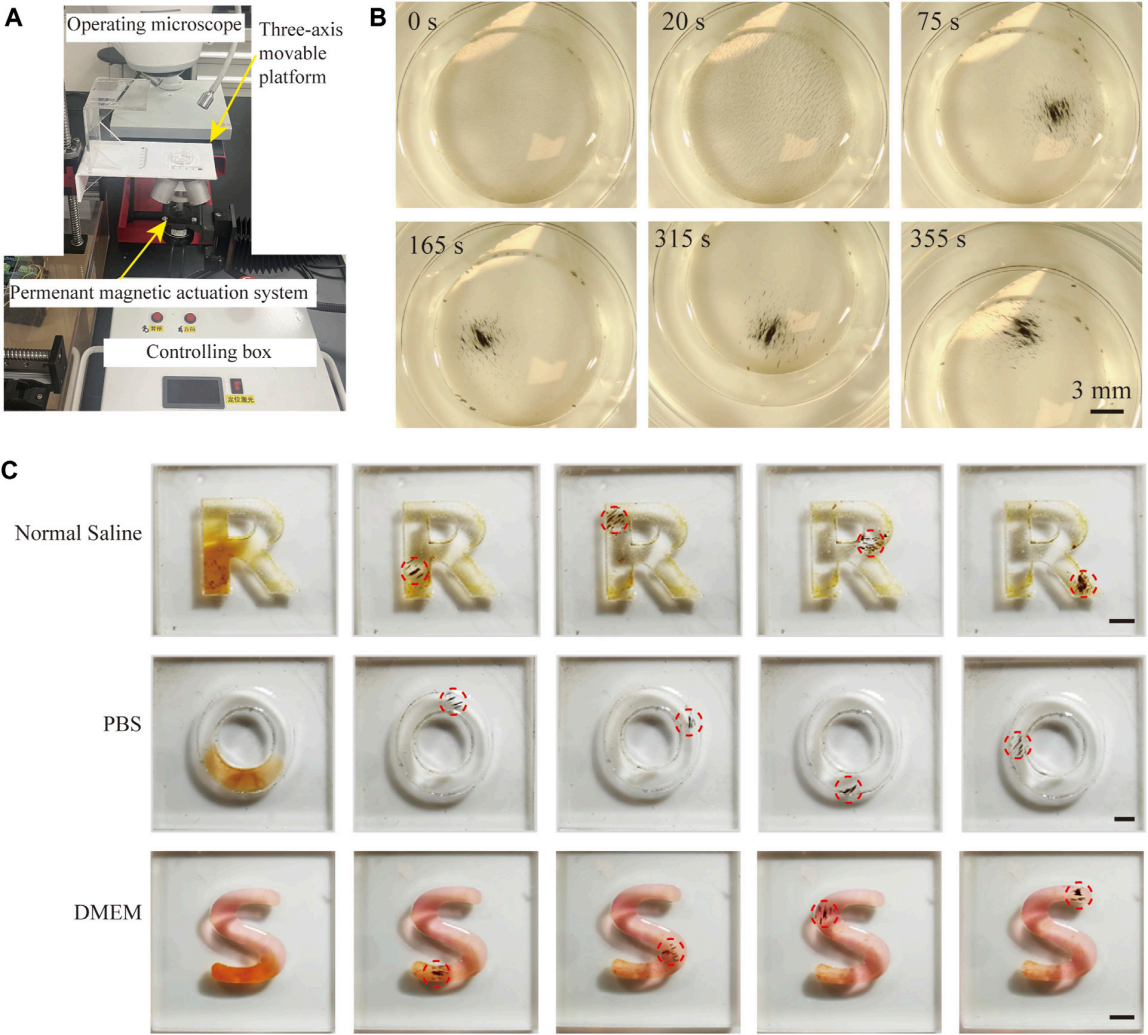
Magnetically actuated locomotion of FSC nanorobot collectives. **(A)** Schematic diagram of the magnetic actuation system. **(B)** Process of magnetically actuated FSC nanorobot aggregation. **(C)** Trajectory locomotion of magnetic FSC nanorobot collectives in normal saline, PBS, and DMEM. Scale bars indicate 3 mm.

Next, to verify that magnetic FSC nanorobots can achieve precise target locomotion, we conducted experiments with a specifically designed permanent magnetic actuation system. The magnetic actuation system consists of four permanent magnets and a rotating motor, which can generate a rotating magnetic field and gradient magnetic field simultaneously (Figure 3A). As shown in Figure 3B, the nanorobots were first arranged into small strips under the action of a static magnetic field after being placed in the magnetic actuation system. When the permanent magnets were rotated, under the combined action of the rotating magnetic field and gradient magnetic field, the magnetic poles of the nanorobot chains attracted each other to aggregate into collectives. In addition, we designed a three-axis movable platform for positioning the samples. By changing the relative position between the platform and the magnetic actuation system, targeted locomotion of the magnetic nanoparticle collectives was achieved.

We further conducted locomotion experiments on magnetic FSC nanorobots in different biological solutions. As shown in Figure 3C, the nanorobot solution was deployed in the ‘R’, ‘O’, and ‘S’ channels containing normal saline, PBS and DMEM, respectively. Under the control of a magnetic field, magnetic FSC nanorobot collectives can achieve trajectory locomotion. All these results indicated that the magnetic FSC nanorobots could achieve target locomotion in biological fluids, providing a foundation for subsequent targeted therapy of tumor cells.

### 3.4 The cellular oxidative stress induced by FSC nanorobot

**FIGURE 4:**
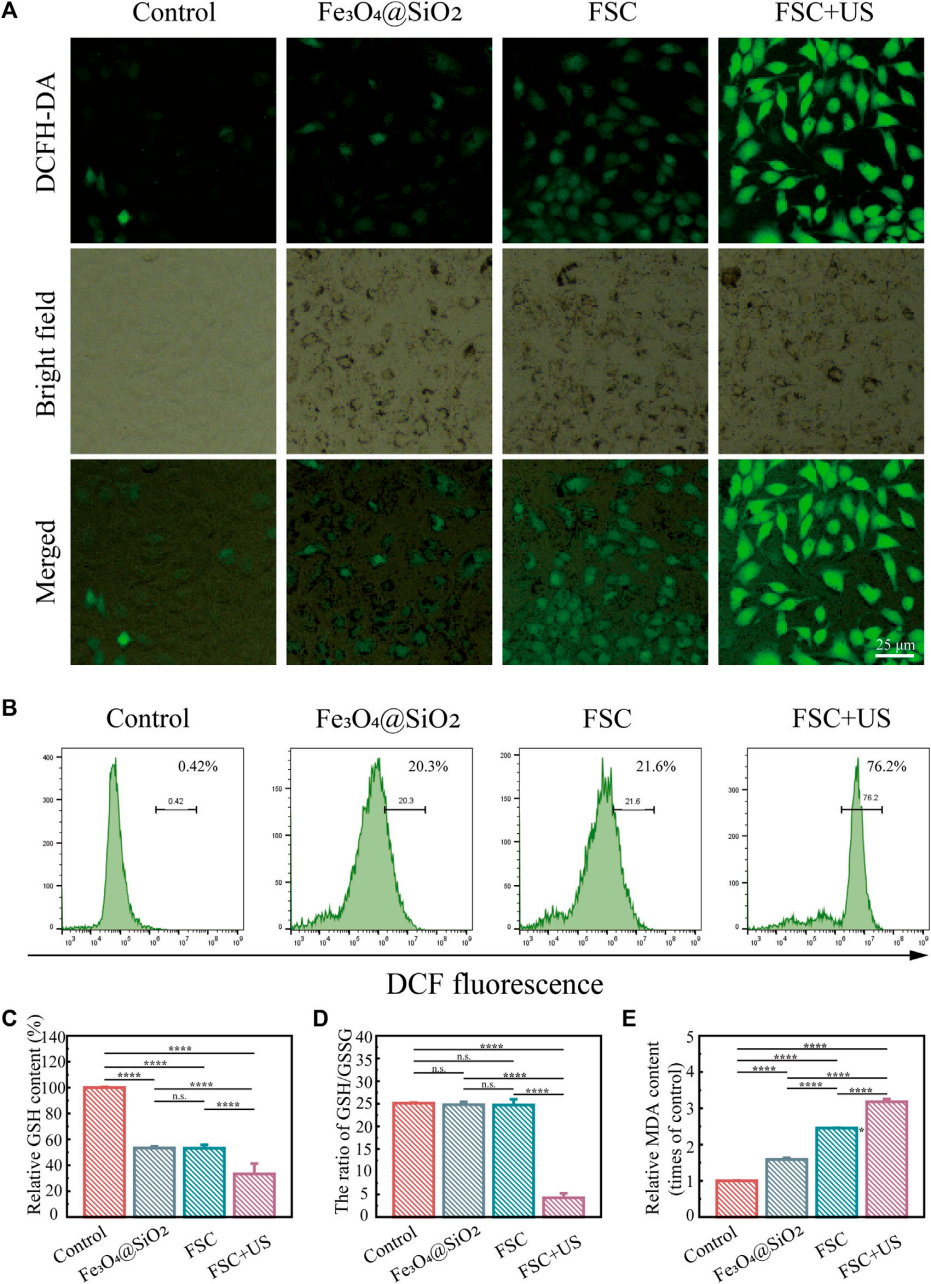
**(A)** Fluorescence images of ID8 cells stained with DCFH-DA after different treatments (control, Fe_3_O_4_@SiO_2_ only, Fe_3_O_4_@SiO_2_-Ce6 (FSC) only, and Fe_3_O_4_@SiO_2_-Ce6 with US irradiation). The scale bars indicate 25 μm. **(B)** Results of quantitative flow cytometry analysis of ROS production in ID8 cells stained with DCFH-DA after different treatments. **(C)** Relative GSH content in ID8 cells after different treatments. **(D)** The ratio of GSH/GSSG. **(E)** Relative MDA content in ID8 cells.

We further examined the production of intracellular ROS after different treatments by fluorescence microscopy and flow cytometry. The fluorescence signal derived from 2′,7′-dichlorofluorescin diacetate (DCFH-DA) was used as a probe of cellular oxidative stress levels. Figure 4A shows minimal fluorescence in the control group, a modest increase in green fluorescence in the Fe_3_O_4_@SiO_2_ and FSC nanorobot groups, which may be due to the role of Fe_3_O_4_ in the Fenton reaction in tumor microenvironment. And green fluorescence was significantly increased in the FSC nanorobot group irradiated with US. This observation was substantiated by the flow cytometry analyses shown in Figure 4B. The fluorescence intensity trend was consistent with that observed by fluorescence microscopy, suggesting the essential role of Ce6 in sonodynamic therapy. Our findings indicate that FSC nanorobots can modulate the tumour microenvironment, elevate ROS levels, and thereby increase therapeutic efficacy.

Glutathione (GSH) and oxidized glutathione (GSSG) are major components of the redox homeostasis system in cells (Lei et al., 2022; Zhu et al., 2021). As displayed in Figure 4C, the GSH levels of the cells in the Fe_3_O_4_@SiO_2_ and FSC groups exhibited a slight decrease, which may be due to the low ROS production deplete the GSH. Moreover, FSC + US significantly reduced the GSH compared with other groups, revealing the efficient GSH depletion ability. The ratio of GSH/GSSG is considered a powerful index of oxidative stress (Wang D. et al., 2023; Liu et al., 2023). The results showed that in the Fe_3_O_4_@SiO_2_ and FSC treatment groups, the GSH/GSSG ratio was slightly varied, while it was greatly decreased in FSC + US group (Figure 4D), altogether confirming the superiority of FSC sonodynamic therapy. Malondialdehyde (MDA) can also be used as a stable marker to evaluate lipid peroxidation (Zhu et al., 2021). The MDA level in each group was opposite to GSH, FSC + US increased significantly (Figure 4E), which illustrates that FSC nanorobots induce sonodynamic therapy associated with lipid peroxidation.

### 3.5 *In vitro* augmented SDT efficacy of FSC nanorobot

**FIGURE 5:**
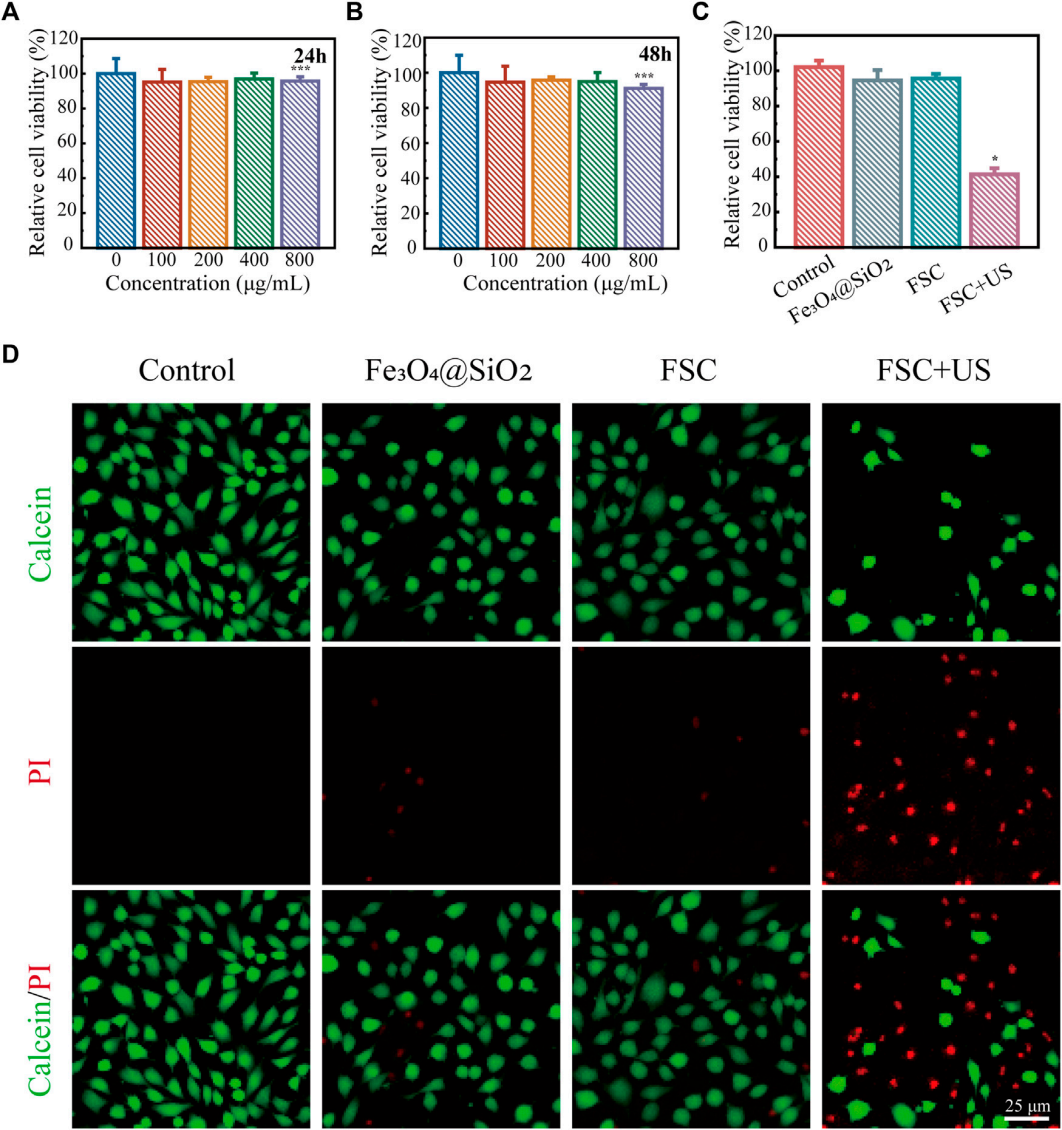
SDT of FSC nanorobot collectives. **(A)** Relative viability of ID8 cells after incubation with high concentrations (0, 100, 200, 400, and 800 μg mL^-1^) of the FSC nanorobots for 24 h and 48 h **(B)**. **(C)** Relative viability of ID8 cells after different treatments, including control (without treatment), Fe_3_O_4_@SiO_2_ only, Fe_3_O_4_@SiO_2_-Ce6 (FSC) only, and Fe_3_O_4_@SiO_2_-Ce6combined with US irradiation. **(D)** Fluorescence images of ID8 cells after different treatments, stained with PI (red fluorescence) and calcein-AM (green fluorescence). The scale bars indicate 25 μm.

Inspired by the excellent ability of the FSC nanorobot to generate ROS under low-intensity ultrasound excitation, we next evaluated the biosafety and the *in vitro* SDT efficacy of the FSC nanorobots in ID8 cells. Initially, the cytotoxicity of the FSC nanorobots was evaluated using the CCK-8 assay. At a concentration of 800 μg mL^-1^, cell viability remained approximately at 95%, highlighting the favorable biocompatibility of FSC nanorobot within the range of 0–800 μg mL^-1^ in ID8 cells (Figures 5A,B). Subsequently, we investigated the efficacy of SDT *in vitro*. The experimental findings demonstrated a decrease in cell viability in the presence of the FSC nanorobot upon US stimulation (Figure 5C). Consistent results were corroborated through calcein-AM/PI staining. Fluorescence microscopy revealed a noteworthy increase in the red signal intensity following US stimulation (Figure 5D). Collectively, these results suggest that after incubation with FSC nanorobots for 24 h, tumour cells can be effectively killed through US stimulation (1.0 MHz, 1.0 Wcm^-2^, 50% duty cycle, 5 min).

### 3.6 Efficacy of magnetic FSC nanorobot for targeted tumor cell therapy

**FIGURE 6:**
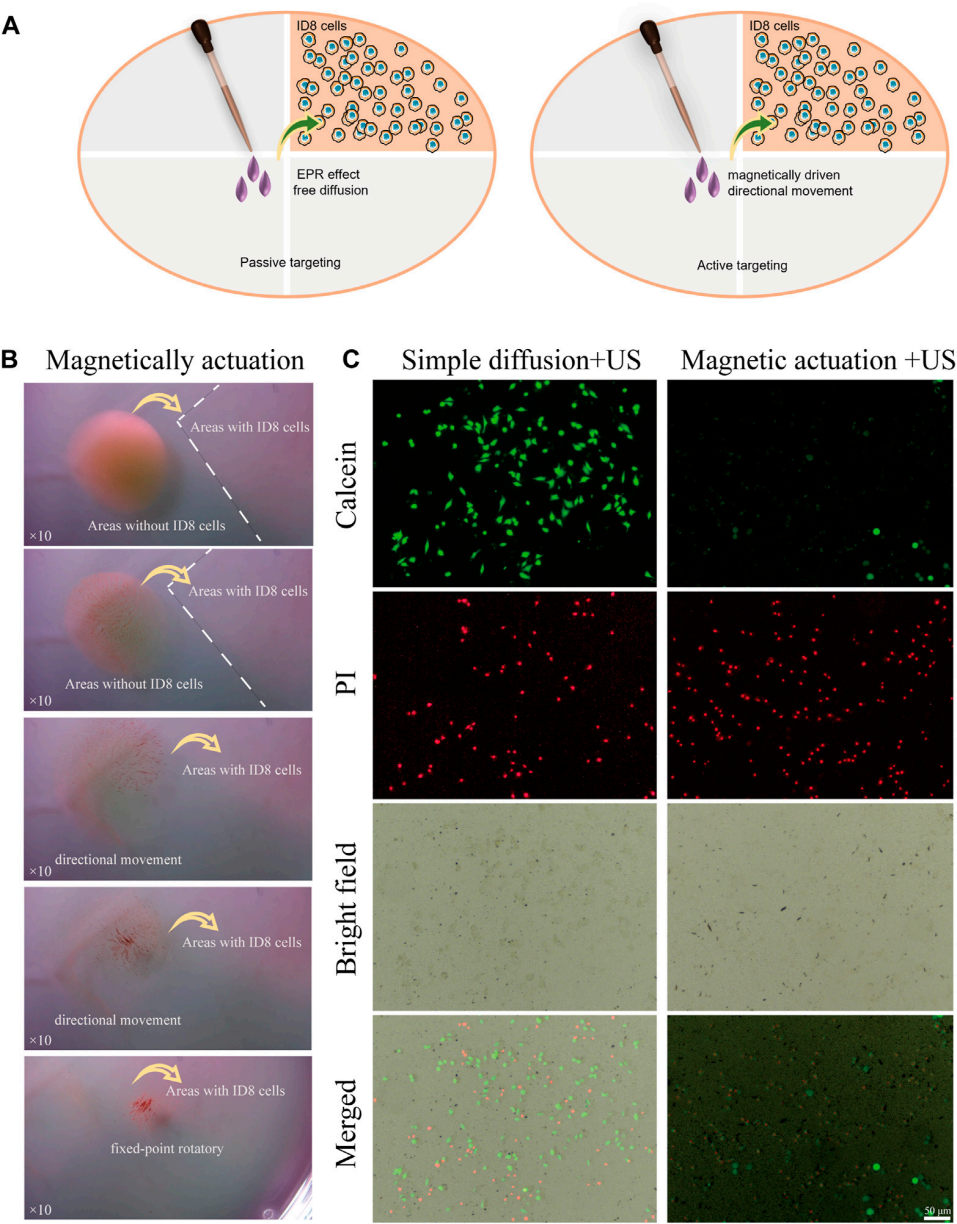
Magnetic FSC nanorobots for targeted tumor cell therapy. **(A)** Schematic illustration of the enhanced SDT effect of magnetic regulation by the FSC nanorobots. **(B)** Process of targeted tumor cell therapy with magnetic FSC nanorobot collectives. **(C)** Fluorescence images of the simple diffusion + US and magnetic actuation + US approaches to targeting FSC nanorobots to tumour cells, which were stained with PI (red fluorescence) and calcein-AM (green fluorescence). The scale bars indicate 50 μm.

We further studied the efficacy of magnetic FSC nanorobots for targeted tumour cell therapy. Two experimental groups were treated with or without magnetic actuation. As shown in Figure 6A, the cell dishes were divided into tumour cell areas and blank areas. The FSC nanorobot dispersion was added to the blank area. In the control group, no magnetic field was applied, and the nanorobots relied on passive diffusion to reach the tumour cell area, simulating the traditional systemic drug administration strategy. For the magnetic actuation group, after the FSC nanorobot dispersion was added to the blank area, a magnetic field was applied to aggregate the nanorobots to one point and then direct the nanorobot collectives to the tumour region. Both groups were subsequently subjected to low-intensity ultrasound of the tumour cell area. The results indicated that only a few FSC nanorobots arrived at the tumour region in the control group, while in the magnetic actuation group, nearly all the FSC nanorobots reached the tumour region (Figure 6B). The SDT results further confirmed the enrichment of Ce6 in the tumour area by the magnetic actuation strategy. As shown in Figure 6C, more tumour cell death occurred under US irradiation. The enhanced sonodynamic effect highlights the ability of FSC nanorobots to facilitate targeted drug delivery and increase the therapeutic efficacy of SDT.

## 4 Conclusion

In summary, we have proposed a magnetically actuated targeting strategy to increase the therapeutic efficacy of SDT and applied it to ovarian cancer therapy *in vitro* as a proof of concept. To validate this concept, a magnetic sonodynamic nanorobot coupled with Ce6 was fabricated. *In vitro* targeting experiments demonstrated the controlled movement of FSC nanorobot collectives under a magnetic field, allowing directed enrichment in the target area. This approach facilitated SDT, resulting in the effective eradication of tumor cells under low-intensity US irradiation. However, this study has certain limitations, including the need for further verification of *in vivo* biosafety and the enrichment rates and efficacy of tumor targeting. This study successfully engineered magnetically actuated targeting multifunctional composite nanorobots, achieving increased sonodynamic effects. This strategy provides a new approach for establishing safe and effective targeted therapies for ovarian cancer.

## Data Availability

The datasets presented in this study can be found in online repositories. The names of the repository/repositories and accession number(s) can be found in the article/Supplementary material.

## References

[B1] Chen K. Zhou A. Zhou X. Liu Y. Xu Y. Ning X. (2023). An intelligent cell-derived nanorobot bridges synergistic crosstalk between sonodynamic therapy and cuproptosis to promote cancer treatment. Nano Lett. 23 (7), 3038–3047. 10.1021/acs.nanolett.3c00434 36951267

[B2] Coleman R. L. Monk B. J. Sood A. K. Herzog T. J. (2013). Latest research and treatment of advanced-stage epithelial ovarian cancer. Nat. Rev. Clin. Oncol. 10 (4), 211–224. 10.1038/nrclinonc.2013.5 23381004 PMC3786558

[B3] Di Donato V. Kontopantelis E. Aletti G. Casorelli A. Piacenti F. Benedetti P. (2017). Trends in mortality after primary cytoreductive surgery for ovarian cancer: a systematic review and metaregression of randomized clinical trials and observational studies. Ann. Surg. Oncol. 24 (6), 1688–1697. 10.1245/s10434-016-5680-7 27896508

[B4] Durno K. Powell M. E. (2022). The role of radiotherapy in ovarian cancer. Int. J. Gynecol. Cancer 32 (3), 366–371. 10.1136/ijgc-2021-002462 35256425

[B5] Gerestein C. G. Damhuis R. A. de Vries M. Reedijk A. Burger C. W. Kooi G. S. (2009). Causes of postoperative mortality after surgery for ovarian cancer. Eur. J. Cancer 45 (16), 2799–2803. 10.1016/j.ejca.2009.06.004 19615887

[B6] Giovanni D. L. Giuseppe R. Maria S. G. Federico R. Stefania B. (2018). Imaging and therapy of ovarian cancer: clinical application of nanoparticles and future perspectives. Theranostics 8 (16), 4279–4294. 10.7150/thno.26345 30214620 PMC6134923

[B7] Guo J. Pan X. Wang C. Liu H. (2022). Molecular imaging-guided sonodynamic therapy. Bioconjug Chem. 33 (6), 993–1010. 10.1021/acs.bioconjchem.1c00288 34595922

[B8] Guo W. Wang T. Huang C. Ning S. Qian H. Wang A. (2023). Platelet membrane-coated C-TiO_2_ hollow nanospheres for combined sonodynamic and alkyl-radical cancer therapy. Nano Res. 16, 782–791. 10.1007/s12274-022-4646-2

[B9] Hu M. Ge X. Chen X. Mao W. Qian X. Yuan W. E. (2020). Micro/nanorobot: a promising targeted drug delivery system. Pharmaceutics 12 (7), 665. 10.3390/pharmaceutics12070665 32679772 PMC7407549

[B10] Kengsakul M. Nieuwenhuyzen-de Boer G. M. Udomkarnjananun S. Kerr S. J. Niehot C. D. van Beekhuizen H. J. (2022). Factors predicting postoperative morbidity after cytoreductive surgery for ovarian cancer: a systematic review and meta-analysis. J. Gynecol. Oncol. 33 (4), e53. 10.3802/jgo.2022.33.e53 35712967 PMC9250852

[B11] Lei G. Zhuang L. Gan B. (2022). Targeting ferroptosis as a vulnerability in cancer. Nat. Rev. Cancer 22 (7), 381–396. 10.1038/s41568-022-00459-0 35338310 PMC10243716

[B12] Li J. Ávila B. E.-F. d. Gao W. Zhang L. Wang J. (2017). Micro/nanorobots for biomedicine: delivery, surgery, sensing, and detoxification. Sci. Robot. 2 (4), eaam6431. 10.1126/scirobotics.aam6431 31552379 PMC6759331

[B13] Liu C. Shen Y. Cavdar O. Huang J. Fang H. (2023). Angiotensin II-induced vascular endothelial cells ferroptosis via P53-ALOX12 signal axis. Clin. Exp. Hypertens. (New York, N.Y.) 45 (1), 2180019. 10.1080/10641963.2023.2180019 36860117

[B14] Ma X. Wang L. Wang P. Liu Z. Cai X. Zheng Y. (2022). An electromagnetically actuated magneto-nanozyme mediated synergistic therapy for destruction and eradication of biofilm. Chem. Eng. J. 431, 133971. 10.1016/j.cej.2021.133971

[B15] McHale A. P. Callan J. F. Nomikou N. Fowley C. Callan B. (2016). Sonodynamic therapy: concept, mechanism and application to cancer treatment. Adv. Exp. Med. Biol. 880, 429–450. 10.1007/978-3-319-22536-4_22 26486350

[B16] Nguyen V. D. Park J. O. Choi E. (2023). Macrophage-based microrobots for anticancer therapy: recent progress and future perspectives. Biomimetics (Basel) 8 (7), 553. 10.3390/biomimetics8070553 37999194 PMC10669771

[B17] Ning S. Dai X. Tang W. Guo Q. Yao X. Wang X. (2022). Cancer cell membrane-coated C-TiO_2_ hollow nanoshells for combined sonodynamic and hypoxia-activated chemotherapy. Acta Bio 152, 562–574. 10.1016/j.actbio.2022.08.067 36067874

[B18] Pan X. Wang H. Wang S. Sun X. Wang L. Liu H. (2018). Sonodynamic therapy (SDT): a novel strategy for cancer nanotheranostics. Sci. China Life Sci. 61, 415–426. 10.1007/s11427-017-9262-x 29666990

[B19] Qian X. Zheng Y. Chen Y. (2016). Micro/nanoparticle-augmented sonodynamic therapy (SDT): breaking the depth shallow of photoactivation. Adv. Mater 28 (37), 8097–8129. 10.1002/adma.201602012 27384408

[B20] Schmidt C. K. Medina-Sanchez M. Edmondson R. J. Schmidt O. G. (2020). Engineering microrobots for targeted cancer therapies from a medical perspective. Nat. Commun. 11 (1), 5618. 10.1038/s41467-020-19322-7 33154372 PMC7645678

[B21] Shi J. Kantoff P. W. Wooster R. Farokhzad O. C. (2017). Cancer nanomedicine: progress, challenges and opportunities. Nat. Rev. Cancer 17 (1), 20–37. 10.1038/nrc.2016.108 27834398 PMC5575742

[B22] Son S. Kim J. H. Wang X. Zhang C. Wu J. Kim J. S. (2020). Multifunctional sonosensitizers in sonodynamic cancer therapy. Chem. Soc. Rev. 49 (11), 3244–3261. 10.1039/c9cs00648f 32337527

[B23] Srivastava S. K. Ahmad A. Miree O. Patel G. K. Rocconi R. P. Singh A. P. (2017). Racial health disparities in ovarian cancer: not just black and white. J. Ovarian Res. 10 (1), 58. 10.1186/s13048-017-0355-y 28931403 PMC5607508

[B24] Sun X. Dong B. Xu H. Xu S. Zhang X. Song H. (2017). Amphiphilic silane modified multifunctional nanoparticles for magnetically targeted photodynamic therapy. ACS Appl. Mater Interfaces 9 (13), 11451–11460. 10.1021/acsami.7b00647 28233996

[B25] Sung H. Ferlay J. Siegel R. L. Laversanne M. Soerjomataram I. Jemal A. (2021). Global cancer statistics 2020: GLOBOCAN estimates of incidence and mortality worldwide for 36 cancers in 185 countries. CA Cancer J. Clin. 71 (3), 209–249. 10.3322/caac.21660 33538338

[B26] Tang X. Manamanchaiyaporn L. Zhou Q. Huang C. Yan X. Zheng Y. (2022). Synergistic integration and pharmacomechanical function of enzyme-magnetite nanoparticle swarms for low-dose fast thrombolysis. Small 18 (34), e2202848. 10.1002/smll.202202848 35905497

[B27] Torre L. A. Trabert B. DeSantis C. E. Miller K. D. Jemal A. Siegel R. L. (2018). Ovarian cancer statistics, 2018. CA Cancer J. Clin. 68 (4), 284–296. 10.3322/caac.21456 29809280 PMC6621554

[B28] Wang D. Zhang M. Zhang Y. Qiu G. Zhang K. Luo T. (2022). Intraparticle double-scattering-decoded sonogenetics for augmenting immune checkpoint blockade and CAR-T therapy. Adv. Sci. Weinh. 9 (32), e2203106. 10.1002/advs.202203106 36156442 PMC9661857

[B29] Wang D. Zhu X. Wang X. Wang Q. Zhang K. Liu Y. (2023). Multichannel sonocatalysis amplifiers target IDH1‐mutated tumor plasticity and attenuate ros tolerance to repress malignant cholangiocarcinoma. Adv. Funct. Mater 33 (48), 2303869. 10.1002/adfm.202303869

[B30] Wang J. Zeng L. Wu N. Liang Y. Jin J. Fan M. (2023). Inhibition of phosphoglycerate dehydrogenase induces ferroptosis and overcomes enzalutamide resistance in castration-resistant prostate cancer cells. Drug Resist. Updat. Rev. Comment. Antimicrob. anticancer Chemother. 70, 100985. 10.1016/j.drup.2023.100985 37423117

[B31] Wang L. Meng Z. Chen Y. Zheng Y. (2021a). Engineering magnetic micro/nanorobots for versatile biomedical applications. Adv. Intel. Syst. 3 (7), 2000267. 10.1002/aisy.202000267

[B32] Wang L. Wang J. Hao J. Dong Z. Liu Z. Zheng Y. (2021d). Guiding drug through interrupted bloodstream for potentiated thrombolysis by C-shaped magnetic actuation system *in vivo* . Adv. Mat. 33 (51), e2105351. 10.1002/adma.202105351 34647345

[B33] Wang L. Zou W. Shen J. Yang S. Wu J. Ying T. (2023). Dual-functional laser-guided magnetic nanorobot collectives against gravity for on-demand thermo-chemotherapy of peritoneal metastasis. Adv. Healthc. Mat. 2303361. 10.1002/adhm.202303361 38115718

[B34] Wang S. Zeng N. Zhang Q. Chen M. Huang Q. (2022). Nanozyme hydrogels for self-augmented sonodynamic/photothermal combination therapy. Front. Oncol. 12, 888855. 10.3389/fonc.2022.888855 35860592 PMC9289279

[B35] Wang X. Wang X. Yue Q. Xu H. Cheng L. Sun L. (2021c). Liquid exfoliation of TiN nanodots as novel sonosensitizers for photothermal-enhanced sonodynamic therapy against cancer. Nano Today 39, 101170. 10.1016/j.nantod.2021.101170

[B36] Wang X. Wang X. Zhong X. Li G. Cheng L. Gong Y. (2020). V-TiO_2_ nanospindles with regulating tumor microenvironment performance for enhanced sonodynamic cancer therapy. Appl. Phys. Rev. 7, 041411. 10.1063/5.0027606

[B37] Wang X. Zhong X. Li J. Liu Z. Cheng L. (2021b). Inorganic nanomaterials with rapid clearance for biomedical applications. Chem. Soc. Rev. 50 (15), 8669–8742. 10.1039/d0cs00461h 34156040

[B38] Wilhelm S. Tavares A. J. Dai Q. Ohta S. Chan W. C. W. Dvorak H. F. (2016). Analysis of nanoparticle delivery to tumours. Nat. Rev. Mater 1 (5), 16014. 10.1038/natrevmats.2016.14

[B39] Yan P. Liu L. H. Wang P. (2020). Sonodynamic therapy (SDT) for cancer treatment: advanced sensitizers by ultrasound activation to injury tumor. ACS Appl. Bio Mater 3 (6), 3456–3475. 10.1021/acsabm.0c00156 35025218

[B40] Yang Y. Wang X. Qian H. Cheng L. (2021). Titanium-based sonosensitizers for sonodynamic cancer therapy. Appl. Mater Today 25, 101215. 10.1016/j.apmt.2021.101215

[B41] Zhang H. Chen J. Zhu X. Ren Y. Zhang H. Zhang Z. (2018). Ultrasound induced phase-transition and invisible nanobomb for imaging-guided tumor sonodynamic therapy. J. Mater Chem. B 6 (38), 6108–6121. 10.1039/c8tb01788c 32254821

[B42] Zhang Y. Yong L. Luo Y. Ding X. Pu D. Zou J. (2019). Enhancement of HIFU ablation by sonosensitizer-loading liquid fluorocarbon nanoparticles with pre-targeting in a mouse model. Sci. Rep. 9 (1), 6982. 10.1038/s41598-019-43416-y 31061456 PMC6502828

[B43] Zhao Y. Xiong H. Li Y. Gao W. Cai X. Zheng Y. (2022). Magnetically actuated reactive oxygen species scavenging nano‐robots for targeted treatment. Adv. Intel. Syst. 4 (7). 10.1002/aisy.202200061

[B44] Zhou H. Mayorga-Martinez C. C. Pane S. Zhang L. Pumera M. (2021). Magnetically driven micro and nanorobots. Chem. Rev. 121 (8), 4999–5041. 10.1021/acs.chemrev.0c01234 33787235 PMC8154323

[B45] Zhu M. Wu P. Li Y. Zhang L. Zong Y. Wan M. (2022). Synergistic therapy for orthotopic gliomas via biomimetic nanosonosensitizer-mediated sonodynamic therapy and ferroptosis. Biomater. Sci. 10 (14), 3911–3923. 10.1039/d2bm00562j 35699471

[B46] Zhu Y. Wu J. Wang K. Xu H. Qu M. Gao Z. (2021). Facile and sensitive measurement of GSH/GSSG in cells by surface-enhanced Raman spectroscopy. Talanta 224, 121852. 10.1016/j.talanta.2020.121852 33379068

